# Association of dietary acid load with diabetes and glucose metabolism index in Chinese adults: a cross-sectional study

**DOI:** 10.29219/fnr.v70.13470

**Published:** 2026-03-20

**Authors:** Shengqi Jia, Yuqin Shi, Xiang Ma, Qiuyin Chen, Weijia Huang, Yulan Zeng, Ping Wang

**Affiliations:** 1 Department of Respiratory and Critical Care Medicine, Liyuan Hospital, Tongji Medical College, Huazhong University of Science and Technology, Wuhan, China; 2 Department of Endocrinology, Institute of Geriatric Medicine, Liyuan Hospital, Tongji Medical College, Huazhong University of Science and Technology, Wuhan, China; 3 Department of Geriatrics, Liyuan Hospital, Tongji Medical College, Huazhong University of Science and Technology, Wuhan, China

**Keywords:** dietary acid load, diabetes, insulin resistance, China Health and Nutrition Survey (CHNS), National Health and Nutrition Examination Survey (NHANES)

## Abstract

**Background:**

Dietary acid load (DAL) has been proven to be associated with hypertension, chronic kidney disease, gout, and the prevalence of type 2 diabetes in several countries. However, its relationship with the prevalence of prediabetes and diabetes in the Chinese population, as well as with fasting blood glucose, fasting insulin levels, and insulin resistance-related indicators, remains unclear.

**Method:**

This is a cross-sectional study based on the China Health and Nutrition Survey (CHNS), which uses Potential Renal Acid Load (PRAL) and Net Endogenous Acid Production (NEAP) to assess DAL. Logistic regression was employed to analyze the relationship between DAL and prediabetes as well as diabetes. Linear regression was used to examine the associations between DAL and fasting blood glucose, fasting insulin levels, estimated glucose disposal rate (eGDR), Homeostasis Model Assessment of Insulin Resistance (HOMA-IR), and the TyG index in the affected population. Restricted cubic spline (RCS) curves were utilized to explore potential nonlinear relationships, and mediation analysis was conducted to investigate the mediating role of insulin resistance in the effects of DAL on fasting blood glucose and insulin. Finally, the findings were validated and compared using data from the National Health and Nutrition Examination Survey (NHANES).

**Results:**

Higher PRAL (odds ratio [OR]: 1.004, 95% confidence interval [CI]: 1.002–1.006) and NEAP (OR: 1.009, 95% CI: 1.005–1.012) were associated with an increased prevalence of diabetes and prediabetes. Elevated levels of PRAL and NEAP were also correlated with higher fasting blood glucose levels and a lower eGDR. Moreover, eGDR played a significant mediating role in the effect of DAL on fasting blood glucose (PRAL: 69.74%, *P* = 0.048; NEAP: 65.75%, *P* = 0.004). However, this phenomenon was not significant in the US population, indicating differences between Chinese and American populations.

**Conclusion:**

High DAL is significantly associated with an increased prevalence of diabetes and prediabetes in the Chinese population, and it influences fasting blood glucose levels in affected individuals by reducing the eGDR. These findings highlight the clinical importance of regulating acid-producing diets to help manage blood glucose levels in individuals with diabetes.

## Popular scientific summary

- At present, there is a lack of research on the relationship between dietary acid load and diabetes and insulin resistance in the Chinese population.
- For the Chinese population, a higher dietary acid load is associated with an increased prevalence of diabetes and prediabetes as well as elevated fasting blood glucose.
- Among them, eGDR demonstrated a significant role in the influence of dietary acids on fasting blood glucose.

Diabetes, mainly caused by insufficient insulin production or secretion or insulin resistance (IR) in peripheral tissues, is a complex disease with strong genetic and environmental susceptibilities (1, 2). The prevalence of diabetes and prediabetes worldwide is still increasing year by year (3, 4).The International Diabetes Federation estimates that currently there are approximately 589 million people worldwide suffering from diabetes, and this number is projected to increase to 853 million by 2025 (5). As of 2023, the number of patients with diabetes aged 20 years and above in China has reached an astonishing 233 million, an increase of approximately 163% compared to 2005, making China the country with the largest number of patients with diabetes in the world (6). Obesity, especially abdominal obesity, is a major risk factor for diabetes and its related comorbidities, including cardiovascular diseases, chronic kidney diseases, and nonalcoholic fatty liver disease (7). Compared with Europeans, Asians have lower lean muscle mass and relatively higher visceral fat content (8). Moreover, the overweight and obesity rates among the Chinese population have been on a continuous upward trend over the past 20 years (9). At the same time, lifestyle factors such as smoking (10), drinking alcohol (11), increased sedentary time, reduced physical activity time (12), decreased sleep time, and unhealthy eating habits are also important risk factors for diabetes (13).

Dietary intake significantly affects the body’s acid‑base balance (14). In epidemiology, potential renal acid load (PRAL) and net endogenous acid production (NEAP) are typically used to assess dietary acid load (DAL). Studies have shown that high DAL can lead to chronic tissue metabolic acidosis, which may in turn promote insulin resistance and type 2 diabetes (T2M) (15–17). A study involving three cohorts in the United States showed that a higher diet-dependent acid load was associated with an increased risk of type 2 diabetes (18). A prospective study in Japan also indicated that a high DAL score was associated with an increased risk of type 2 diabetes in Japanese men (19). However, a study in Sweden showed that acid load scores were not associated with the incidence of diabetes (20). Therefore, the relationship between low-grade acidosis caused by diet and insulin resistance remains controversial.

At present, there is still a lack of research on the impact of DAL on diabetes and insulin resistance in the Chinese population. Therefore, this study aims to verify and expand on previous research findings, explore the relationship between DAL and diabetes in the Chinese population through the Chinese Nutrition and Health Survey (CHNS), and examine whether acid load scores are related to intermediate traits of diabetes.

## Materials and methods

### Data collection and participants

**Fig. 1:**
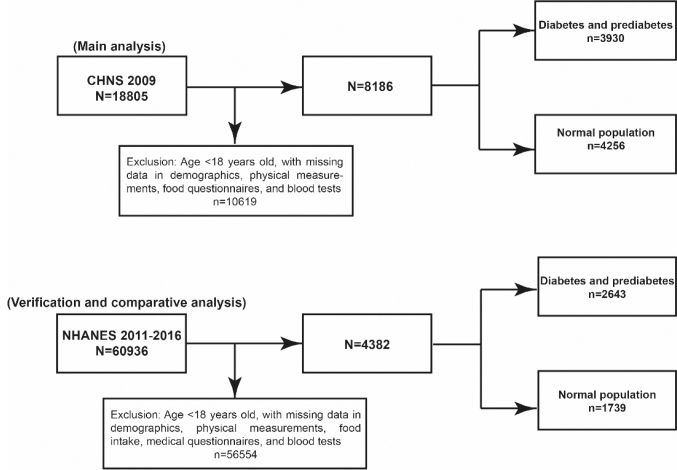
The flow chart.

This study utilized data from the CHNS and the National Health and Nutrition Survey (NHANES) in the United States. The CHNS was initiated in 1989 and conducts follow-up surveys every 2–3 years, involving a total of 7,200 households in 15 provinces and municipalities directly under the Central Government (21). Biological sample information was only collected in 2009, so this study uses CHNS data from 2009 for research. The inclusion and exclusion criteria are as follows: 1) Select the CHNS data of 2009; 2) Exclude the population with incomplete or missing demographic variables, including: age (<18 years old), gender, educational attainment, and place of residence; 3) Exclude individuals with general physical conditions and deficiencies in related blood markers, including: height, weight, waist circumference, smoking questionnaire, drinking questionnaire, fasting blood glucose, fasting insulin, glycated hemoglobin, uric acid, creatinine, systolic blood pressure, and diastolic blood pressure; 4) Exclude the population with missing disease questionnaires and dietary data, including the average intake values of myocardial infarction, stroke, energy, fat, cholesterol, and carbohydrates. A total of 8,186 participants were included for analysis, among whom 3,930 were patients with diabetes and prediabetes. The National Health and Nutrition Examination Survey (NHANES) is a series of cross-sectional, complex, multistage surveys conducted by the Centers for Disease Control and Prevention (CDC) on a nationally representative sample of the noninstitutionalized U.S. population. It provides comprehensive data on the health and nutritional status of participants. This study utilized data from three NHANES cycles (2011–2016). After excluding records with missing demographic information, relevant disease questionnaires, physical examinations, and biomarker data, a total of 4,382 participants were included in the analysis, among whom 2,643 were diagnosed with diabetes or prediabetes. The detailed inclusion and exclusion process is illustrated in Fig. 1.

All participants provided informed consent. The data from the CHNS were approved by the Institutional Review Boards at the University of North Carolina at Chapel Hill and the China National Institute of Nutrition and Health. The NHANES data were approved by the Ethics Review Board of the National Center for Health Statistics (NCHS). This study was conducted in full accordance with the ethical principles established by the Declaration of Helsinki.

### DAL estimations

The DAL was calculated using the PRAL and NEAP formulas established by Remer and Manz, as well as Frassetto and colleagues (22). The formulas are as follows:

PRAL (mEq/d) = 0.4888 × protein intake (g/d) + 0.0366 × phosphorus (mg/d) − 0.0205 × potassium (mg/d) − 0.0125 × calcium (mg/d) − 0.0263 × magnesium (mg/d).

NEAP (mEq/d) = (54.5 × protein intake (g/d) ÷ potassium intake (mEq/d)) − 10.2.

The CHNS collected dietary intake data from participants over 3 consecutive days, including 2 weekdays and 1 weekend day, using 24-h dietary recall questionnaires (21). Nutrient and energy intake for each food item were calculated based on the Chinese Food Composition Table (2002 and 2004 editions). The average intake across the 3 days was used for analysis. The validity and clinical applicability of the CHNS dietary recall method have been described in previous literature (23, 24).

Dietary intake information in NHANES was obtained directly from the dietary interview component. Nutrient and total energy intake for all participants were estimated using a computer-assisted 24-h dietary recall method. The average intake from the first and second days was used for analysis. The dietary interviews were conducted as part of the ‘What We Eat in America’ (WWEIA) program, a collaborative effort between the U.S. Department of Health and Human Services (DHHS) and the U.S. Department of Agriculture (USDA). The validity and clinical applicability of the NHANES dietary recall methodology have been well documented in previous literature (25, 26).

### Definition of diabetes and prediabetes

In the current study, we primarily assessed blood glucose status based on the ADA standards. Diabetes was defined as self-reported diagnosis by a physician or healthcare professional, hemoglobin A1c (HbA1c) ≥ 6.5%, fasting plasma glucose (FPG) ≥ 126 mg/dL, or a 2-h oral glucose tolerance test (OGTT) value ≥ 200 mg/dL.

Prediabetes was defined as HbA1c ranging from 5.7 to 6.4%, FPG between 100 and 125 mg/dL, or a 2-h OGTT value between 140 and 199 mg/dL.

Normal glucose metabolism was defined as HbA1c < 5.7%, FPG < 100 mg/dL, and a 2-h OGTT value < 140 mg/dL.

### Definition of insulin resistance indicators

We used the estimated glucose disposal rate (eGDR), homeostatic model assessment of insulin resistance (HOMA-IR), and triglyceride-glucose index (TyG) to assess insulin resistance. The formulas for calculation are as follows:

eGDR = 21.158 − (0.09 × waist circumference [cm]) − (3.407 × hypertension [yes 1 or no 0]) − (0.551 × glycated hemoglobin A1c [HbA1c] [%]) (27);

HOMA-IR = (fasting glucose [mmol/L] × fasting insulin [µU/mL])/22.5 (28);

TyG = Ln [fasting triglyceride (mg/dL) × fasting glucose (mg/dL)/2] (29).

### Definition of covariates

For participants in the CHNS, potential confounders were identified based on existing literature and clinical knowledge. In this study, demographic and social covariates included: age (continuous), gender (male/female), educational level (junior high school and below, technical or vocational school, college and above), and residential area (urban, suburban). Physical measurements and lifestyle factors included: body mass index (BMI) and smoking status (never smoked, former smoker, current smoker). Comorbidities such as hypertension, myocardial infarction, stroke, hyperuricemia, and chronic kidney disease were also included as covariates based on questionnaire data. Dietary energy intake covariates comprised: average intake of energy, fat, cholesterol, and carbohydrates.

For NHANES participants, the covariates included age (continuous), gender (male/female), race/ethnicity (non-Hispanic white, non-Hispanic black, Mexican American, other Hispanic, and other races), educational attainment (less than high school, high school, and more than high school), poverty-income ratio (PIR), BMI, smoking status (never smoked, former smoker, current smoker), and comorbidities including hypertension, coronary heart disease, myocardial infarction, angina, heart failure, stroke, hyperuricemia, and chronic kidney disease. The selection and inclusion of these covariates have been described in previous publications.

### Statistical analysis

Continuous variables are presented as mean (standard deviation, SD) or median (interquartile range, IQR), while categorical variables are summarized as frequencies. Group comparisons were performed using Student’s t-test, Fisher’s exact test, or the chi-square test, as appropriate.

The primary objectives of this study were to investigate whether DAL is associated with the prevalence of diabetes and prediabetes in the Chinese population and to examine the relationship between DAL and fasting blood glucose, fasting insulin, and insulin resistance indices among individuals with diabetes or prediabetes.

First, univariable and multivariable logistic regression analyses were employed to assess the association between DAL and the prevalence of diabetes and prediabetes. Second, univariable and multivariable linear regression models were used to evaluate the relationship between DAL and fasting blood glucose, fasting insulin, and insulin resistance markers in the affected population. Participants were categorized into four groups according to quartiles of DAL scores (Q1–Q4). To control the potential mixing effect, four sequential regression models were constructed: Model 1: Unadjusted. Model 2: Adjusted for demographic variables, including age, gender, educational level, and residential area. Model 3: Further adjusted for BMI, smoking status, hypertension, myocardial infarction, angina, hyperuricemia, and chronic kidney disease. Model 4: Additionally adjusted for average intake of energy, fat, and carbohydrates.

Mediation analysis was conducted to examine the potential mediating effect of insulin resistance indicators on the association between DAL and fasting blood glucose and insulin. Restricted cubic spline (RCS) regression was used to visualize and test for potential nonlinear relationships between DAL and fasting glucose, fasting insulin, and insulin resistance parameters.

Finally, sensitivity analyses were performed to assess the influence of DAL on fasting glucose, fasting insulin, and insulin resistance indices in both normoglycemic individuals and the overall population. A two-sided *P*-value < 0.05 was considered statistically significant. Comparative analyses using NHANES data are provided in Supplementary Materials 2.

## Result

### Baseline characteristics of the participant population

In this study, a total of 8,186 individuals were included. The overall characteristics of the participants are presented in Table 1. Among them, 3,930 participants were identified as having prediabetes or diabetes. Compared to the normoglycemic group, individuals with prediabetes or diabetes had significantly higher mean age, proportion of males, BMI, proportion of former and current smokers, HbA1c, fasting triglycerides, blood glucose, insulin levels, TyG index, PRAL, NEAP, and prevalence of comorbidities including hypertension, myocardial infarction, stroke, chronic kidney disease, and hyperuricemia (all *P* < 0.05). Conversely, they had significantly lower educational attainment, proportion of never-smokers, potassium intake, and eGDR (all *P* < 0.05). No statistically significant differences were observed between the two groups in terms of residential area or intake of phosphorus, calcium, magnesium, calories, carbohydrates, fat, or protein (all *P* > 0.05).

**Table 1:** The population baseline table of CHNS

Variables	Diabetes or prediabetes population			*P*
	Total (*n* = 8,186)	No (*n* = 4,256)	Yes (*n* = 3,930)	
**Age** (years)	50.33 ± 15.00	46.54 ± 15.03	54.45 ± 13.82	**<0.001**
**Sex**				**0.004**
Male	3,813 (46.58)	1,917 (45.04)	1,896 (48.24)	
Female	4,373 (53.42)	2,339 (54.96)	2,034 (51.76)	
**Education**				**<0.001**
Below high school	3,545 (43.33)	1,675 (39.38)	1,870 (47.61)	
High school	4,230 (51.71)	2,335 (54.90)	1,895 (48.24)	
Above high school	406 (4.96)	243 (5.71)	163 (4.15)	
**Place**				0.061
Urban	2,676 (32.69)	1,431 (33.62)	1,245 (31.68)	
Rural	5,510 (67.31)	2,825 (66.38)	2,685 (68.32)	
**BMI**	23.38 ± 3.47	22.65 ± 3.22	24.16 ± 3.55	**<0.001**
**Hypertension**				**<0.001**
No	7,103 (86.77)	3,895 (91.52)	3,208 (81.63)	
Yes	1,083 (13.23)	361 (8.48)	722 (18.37)	
**Myocardial infarction**				**0.012**
No	8,107 (99.03)	4,226 (99.30)	3,881 (98.75)	
Yes	79 (0.97)	30 (0.70)	49 (1.25)	
**Stroke**				**<0.001**
No	8,074 (98.63)	4,226 (99.30)	3,848 (97.91)	
Yes	112 (1.37)	30 (0.70)	82 (2.09)	
**Chronic kidney disease**				**<0.001**
No	7,235 (88.38)	3,892 (91.45)	3,343 (85.06)	
Yes	951 (11.62)	364 (8.55)	587 (14.94)	
**Hyperuricemia**				**<0.001**
No	6,936 (84.73)	3,769 (88.56)	3,167 (80.59)	
Yes	1,250 (15.27)	487 (11.44)	763 (19.41)	
**Smoking**				**0.003**
Former	265 (3.24)	122 (2.87)	143 (3.64)	
Never	5,648 (69.02)	3,005 (70.62)	2,643 (67.29)	
Now	2,270 (27.74)	1,128 (26.51)	1,142 (29.07)	
**P** (mg)	1855.53 ± 688.12	1859.91 ± 691.77	1850.79 ± 684.21	0.549
**K** (mg)	3274.33 ± 1444.29	3313.20 ± 1472.41	3232.24 ± 412.19	**0.011**
**Ca** (mg)	696.70 ± 482.66	693.23 ± 482.41	700.46 ± 82.96	0.498
**Mg** (mg)	541.69 ± 219.25	542.26 ± 221.39	541.07 ± 16.95	0.806
**Calorie** (kcal)	2137.06 ± 663.35	2136.83 ± 635.64	2137.30 ± 692.19	0.975
**Carbohydrate** (g)	294.77 ± 101.93	296.30 ± 101.32	293.11 ± 102.57	0.157
**Fat** (g)	74.92 ± 40.27	74.65 ± 36.10	75.20 ± 44.35	0.542
**Protein** (g)	65.90 ± 22.97	65.68 ± 22.64	66.15 ± 23.32	0.356
**Grains** (g)	902.88 ± 363.40	911.06 ± 371.91	894.02 ± 353.80	**0.034**
**Fruits** (g)	140.01 ± 226.57	146.62 ± 236.77	132.85 ± 214.77	**0.006**
**Starchy Vegetables** (g)	73.90 ± 115.02	77.15 ± 119.40	70.37 ± 109.98	**0.008**
**Legumes** (g)	91.34 ± 131.86	94.03 ± 133.91	88.44 ± 129.56	0.055
**Soy Products** (g)	87.27 ± 117.60	86.11 ± 113.68	88.52 ± 121.70	0.354
**Nuts** (g)	7.43 ± 38.06	7.41 ± 35.04	7.46 ± 41.08	0.957
**Beverages** (g)	4.62 ± 47.04	6.47 ± 57.37	2.62 ± 32.23	**<0.001**
**Sugars** (g)	0.10 ± 2.15	0.11 ± 2.40	0.08 ± 1.84	0.550
**Pickles** (g)	4.41 ± 14.71	4.78 ± 15.30	4.01 ± 14.03	**0.018**
**Meat** (g)	151.37 ± 144.94	159.40 ± 146.79	142.67 ± 142.42	**<0.001**
**Poultry** (g)	30.29 ± 70.24	32.34 ± 71.33	28.07 ± 68.99	**0.006**
**Aquatic Products** (g)	209.77 ± 342.40	240.90 ± 379.48	176.06 ± 293.42	**<0.001**
**Eggs** (g)	349.90 ± 344.14	301.21 ± 315.35	402.63 ± 365.61	**<0.001**
**Dairy Products** (g)	41.46 ± 117.67	37.19 ± 114.22	46.08 ± 121.14	**<0.001**
**Triglyceride** (mg/dL)	148.34 ± 130.63	124.23 ± 97.66	174.46 ± 154.61	**<0.001**
**Glucose** (mg/dL)	97.21 ± 26.30	86.75 ± 8.32	108.53 ± 33.45	**<0.001**
**Insulin** (μIU/mL)	14.40 ± 22.51	11.15 ± 14.17	17.92 ± 28.54	**<0.001**
**HbA1c** (%)	5.62 ± 0.88	5.22 ± 0.37	6.06 ± 1.04	**<0.001**
**TyG**	8.63 ± 0.72	8.40 ± 0.60	8.88 ± 0.76	**<0.001**
**eGDR**	10.16 ± 1.82	10.77 ± 1.47	9.50 ± 1.93	**<0.001**
**HOMA-IR**	3.76 ± 7.18	2.41 ± 2.94	5.23 ± 9.68	**<0.001**
**PRAL**	10.05 ± 22.52	9.33 ± 23.15	10.82 ± 21.78	**0.003**
**NEAP**	35.38 ± 13.15	34.69 ± 12.96	36.13 ± 13.31	**<0.001**

The data are presented as the numbers (%). Significant *P*-values < 0.05 are in bold.

### Association between DAL and diabetes and prediabetes

As shown in Tables 2 and 3, whether DAL was treated as a continuous variable or categorized into quartiles (Q1–Q4), higher acid load levels and the highest quartile (Q4) remained significantly associated with an increased risk of diabetes and prediabetes in the adjusted models (Model 2, Model 3, and Model 4) (*P* < 0.001). It is noteworthy, however, that in the unadjusted Model 1, the results for the continuous DAL variables showed odds ratios (ORs) below 1 (PRAL: 0.995; NEAP: 0.994), while the highest quartile (Q4) exhibited ORs significantly greater than 1 (PRAL: 1.197; NEAP: 1.276), with all results being statistically significant (*P* < 0.05).

**Table 2:** ORs and 95% CI for diabetes and prediabetes risk based on PRAL

	Model 1	Model 2	Model 3	Model 4
**Continuous**	**0.995 (0.992, 0.998) 0.002**	**1.005 (1.003, 1.007) <0.001**	**1.004 (1.002, 1.006) <0.001**	**1.004 (1.002, 1.006) <0.001**
**Q1**	**Ref**	**Ref**	**Ref**	**Ref**
**Q2**	1.043 (0.922, 1.179) 0.501	1.038 (0.913, 1.179) 0.568	1.073 (0.941, 1.223) 0.296	1.069 (0.937, 1.220) 0.318
**Q3**	1.085 (0.959, 1.226) 0.194	**1.142 (1.005, 1.298) 0.042**	**1.159 (1.016, 1.322) 0.028**	**1.152 (1.010, 1.314) 0.035**
**Q4**	**1.197 (1.059, 1.354) 0.004**	**1.323 (1.162, 1.506) <0.001**	**1.299 (1.137, 1.483) <0.001**	**1.287 (1.122, 1.477) <0.001**

Model 1: No covariates were adjusted.

Model 2: Adjusted for age, sex, education, and place.

Model 3: Adjusted for age, sex, education, place, BMI, hypertension, myocardial infarction, stroke, chronic kidney disease, hyperuricemia, and smoke status.

Model 4: Adjusted for age, sex, education, place, BMI, hypertension, myocardial infarction, stroke, chronic kidney disease, hyperuricemia, smoke status, calorie, carbohydrate, and fat.

The meaning of the bolded font is the result with significant statistical significance in the table.

**Table 3:** ORs and 95% CI for diabetes and prediabetes risk based on NEAP

	Model 1	Model 2	Model 3	Model 4
**Continuous**	**0.994 (0.991, 0.997) <0.001**	**1.010 (1.006, 1.014) <0.001**	**1.009 (1.005, 1.013) <0.001**	**1.009 (1.005, 1.012) <0.001**
**Q1**	**Ref**	**Ref**	**Ref**	**Ref**
**Q2**	1.007 (0.890, 1.138) 0.917	1.044 (0.919, 1.187) 0.506	1.067 (0.935, 1.216) 0.336	1.061 (0.930, 1.210) 0.380
**Q3**	1.079 (0.954, 1.219) 0.227	1.117 (0.983, 1.269) 0.089	**1.142 (1.002, 1.302) 0.047**	1.130 (0.990, 1.289) 0.070
**Q4**	**1.267 (1.121, 1.433) <0.001**	**1.346 (1.184, 1.531) <0.001**	**1.345 (1.179, 1.535) <0.001**	**1.326 (1.161, 1.514) <0.001**

Model 1: No covariates were adjusted.

Model 2: Adjusted for age, sex, education, and place.

Model 3: Adjusted for age, sex, education, place, BMI, hypertension, myocardial infarction, stroke, chronic kidney disease, hyperuricemia, and smoke status.

Model 4: Adjusted for age, sex, education, place, BMI, hypertension, myocardial infarction, stroke, chronic kidney disease, hyperuricemia, smoke status, calorie, carbohydrate, and fat.

The meaning of the bolded font is the result with significant statistical significance in the table.

### Association between DAL and measures of blood glucose and insulin resistance among patients

As presented in Tables 4 and 5, after full adjustment for covariates, both continuous PRAL (β: 0.012, 95% confidence interval [CI]: 0.004, 0.020, *P* = 0.035) and NEAP (β: 0.111, 95% CI: 0.033, 0.189, *P* = 0.005) were positively associated with increased fasting blood glucose. Similarly, the highest quartile (Q4) of PRAL (β: 3.135, 95% CI: 0.079, 6.191, *P* = 0.044) and NEAP (β: 3.694, 95% CI: 0.765, 6.623, *P* = 0.013) also showed significant positive associations with elevated fasting glucose.

**Table 4:** The association between PRAL and fasting blood glucose

	Model 1	Model 2	Model 3	Model 4
**Continuous**	**0.050 (0.002, 0.098) 0.042**	**0.051 (0.003, 0.100) 0.038**	0.046 (−0.002, 0.094) 0.061	**0.012 (0.004, 0.100) 0.035**
**Q1**	**Ref**	**Ref**	**Ref**	**Ref**
**Q2**	2.408 (−0.549, 5.365) 0.111	2.253 (−0.690, 5.196) 0.134	2.208 (−0.702, 5.119) 0.137	2.041 (−0.866, 4.948) 0.169
**Q3**	2.938 (−0.020, 5.896) 0.052	**2.992 (0.041, 5.943)** **0.047**	2.857 (−0.059, 5.774) 0.055	**3.076 (0.171, 5.980) 0.038**
**Q4**	2.760 (−0.195, 5.716) 0.067	2.852 (−0.139, 5.842) 0.062	2.389 (−0.569, 5.347) 0.114	**3.135 (0.079, 6.191) 0.044**

Model 1: No covariates were adjusted.

Model 2: Adjusted for age, sex, education, and place.

Model 3: Adjusted for age, sex, education, place, BMI, hypertension, myocardial infarction, stroke, chronic kidney disease, hyperuricemia, and smoke status.

Model 4: Adjusted for age, sex, education, place, BMI, hypertension, myocardial infarction, stroke, chronic kidney disease, hyperuricemia, smoke status, calorie, carbohydrate, and fat.

The meaning of the bolded font is the result with significant statistical significance in the table.

**Table 5:** The association between NEAP and fasting blood glucose

	Model 1	Model 2	Model 3	Model 4
**Continuous**	**0.146 (0.067, 0.224) <0.001**	**0.140 (0.061, 0.218) <0.001**	**0.118 (0.040, 0.196) 0.003**	**0.111 (0.033, 0.189) 0.005**
**Q1**	**Ref**	**Ref**	**Ref**	**Ref**
**Q2**	2.901 (−0.055, 5.857) 0.055	2.858 (−0.088, 5.805) 0.057	2.422 (−0.494, 5.337) 0.104	2.473 (−0.430, 5.376) 0.095
**Q3**	2.930 (−0.026, 5.886) 0.052	2.755 (−0.201, 5.712) 0.068	2.450 (−0.457, 5.376) 0.101	2.687 (−0.233, 5.608) 0.071
**Q4**	**4.458 (1.501, 7.414) 0.003**	**4.445 (1.493, 7.398) 0.003**	**3.947 (1.022, 6.871) 0.008**	**3.694 (0.765, 6.623) 0.013**

Model 1: No covariates were adjusted.

Model 2: Adjusted for age, sex, education, and place.

Model 3: Adjusted for age, sex, education, place, BMI, hypertension, myocardial infarction, stroke, chronic kidney disease, hyperuricemia, and smoke status.

Model 4: Adjusted for age, sex, education, place, BMI, hypertension, myocardial infarction, stroke, chronic kidney disease, hyperuricemia, smoke status, calorie, carbohydrate, and fat.

The meaning of the bolded font is the result with significant statistical significance in the table.

Regarding the relationship between DAL and the eGDR, as shown in Tables 6 and 7, both continuous PRAL (β: −0.002, 95% CI: −0.003, −0.001, *P* = 0.002) and NEAP (β: −0.004, 95% CI: −0.006, −0.002, *P* < 0.001) were inversely associated with eGDR. This negative association was also observed in the highest quartile (Q4) for both PRAL (β: −0.091, 95% CI: −0.170, −0.012, *P* = 0.023) and NEAP (β: −0.105, 95% CI: −0.180, −0.030, *P* = 0.006).

**Table 6:** The association between PRAL and eGDR

	Model 1	Model 2	Model 3	Model 4
**Continuous**	−0.001 (−0.004, 0.001) 0.297	−**0.004 (**−**0.006,** −**0.001) 0.007**	−**0.002 (**−**0.003,** −**0.001) 0.003**	−**0.002 (**−**0.003,** −**0.001) 0.002**
**Q1**	**Ref**	**Ref**	**Ref**	**Ref**
**Q2**	−0.122 (−0.283, 0.058) 0.196	−0.105 (−0.268, 0.059) 0.209	−**0.096 (**−**0.171,** −**0.022) 0.011**	−**0.097 (**−**0.172,** −**0.022) 0.011**
**Q3**	−0.063 (−0.234, 0.107) 0.468	−0.132 (−0.296, 0.331) 0.113	−0.057 (−0.132, 0.018) 0.136	−0.060 (−0.135, 0.015) 0.115
**Q4**	−0.008 (−0.178, 0.163) 0.929	−0.161 (−0.327, 0.004) 0.056	**−0.087 (−0.163, −0.011) 0.024**	**−0.091 (−0.170, −0.012) 0.023**

Model 1: No covariates were adjusted.

Model 2: Adjusted for age, sex, education, and place.

Model 3: Adjusted for age, sex, education, place, BMI, hypertension, myocardial infarction, stroke, chronic kidney disease, hyperuricemia, and smoke status.

Model 4: Adjusted for age, sex, education, place, BMI, hypertension, myocardial infarction, stroke, chronic kidney disease, hyperuricemia, smoke status, calorie, carbohydrate, and fat.

The meaning of the bolded font is the result with significant statistical significance in the table.

**Table 7:** The association between NEAP and eGDR

	Model 1	Model 2	Model 3	Model 4
**Continuous**	**−0.008 (−0.013, −0.004) <0.001**	**−0.008 (−0.013, −0.004) <0.001**	**−0.004 (−0.99, −0.002) <0.001**	**−0.004 (−0.99, −0.002) <0.001**
**Q1**	**Ref**	**Ref**	**Ref**	**Ref**
**Q2**	−0.117 (−0.288, 0.053) 0.178	−0.144 (−0.308, 0.019) 0.083	−0.055 (−0.130, 0.020) 0.150	−0.056 (−0.131, 0.019) 0.141
**Q3**	−0.121 (−0.292, 0.049) 0.163	**−0.179 (−0.342, −0.015) 0.033**	−0.097 (−0.172, −0.022) 0.011	**−0.102 (−0.177, −0.026) 0.008**
**Q4**	**−0.193 (−0.363, −0.022) 0.027**	**−0.242 (−0.406, −0.079) 0.004**	**−0.104 (−0.179, −0.029) 0.006**	**−0.105 (−0.180, −0.030) 0.006**

Model 1: No covariates were adjusted.

Model 2: Adjusted for age, sex, education, and place.

Model 3: Adjusted for age, sex, education, place, BMI, hypertension, myocardial infarction, stroke, chronic kidney disease, hyperuricemia, and smoke status.

Model 4: Adjusted for age, sex, education, place, BMI, hypertension, myocardial infarction, stroke, chronic kidney disease, hyperuricemia, smoke status, calorie, carbohydrate, and fat.

The meaning of the bolded font is the result with significant statistical significance in the table.

Meanwhile, among individuals with diabetes or prediabetes, no significant associations were found between DAL and fasting insulin levels, the TyG index, or HOMA-IR (see Supplementary Tables 1–6) (*P* > 0.05).

### Intermediary analysis

**Fig. 2:**
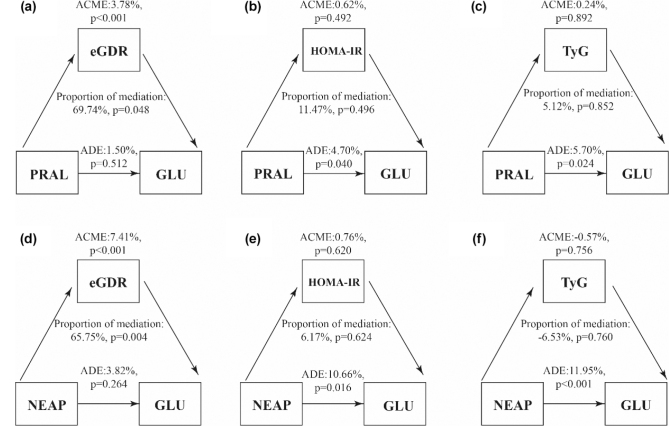
Analysis of mediating effects. (a) The mediating effect of eGDR between PRAL and fasting blood glucose was 65.74%, which was significant. (b) The mediating effect of HOMA-IR in PRAL and fasting blood glucose was 11.47%, which was not significant. (c) The mediating effect of TyG in PRAL and fasting blood glucose was 5.12%, which was not significant. (d) The mediating effect of eGDR between NEAP and fasting blood glucose was 65.75%, which was significant. (e) The mediating effect of HOMA-IR in NEAP and fasting blood glucose was 6.17%, which was not significant. (f) The mediating effect of TyG in NEAP and fasting blood glucose was −6.53%, which was not significant.

As shown in Fig. 2, eGDR exhibited a significant mediating effect in the association between PRAL and fasting blood glucose, accounting for 69.74% of the total effect (*P* = 0.048). Similarly, eGDR also mediated 65.75% of the effect of NEAP on fasting blood glucose (*P* = 0.004). In contrast, no significant mediating effects were observed for HOMA-IR or the TyG index.

**Fig. 3:**
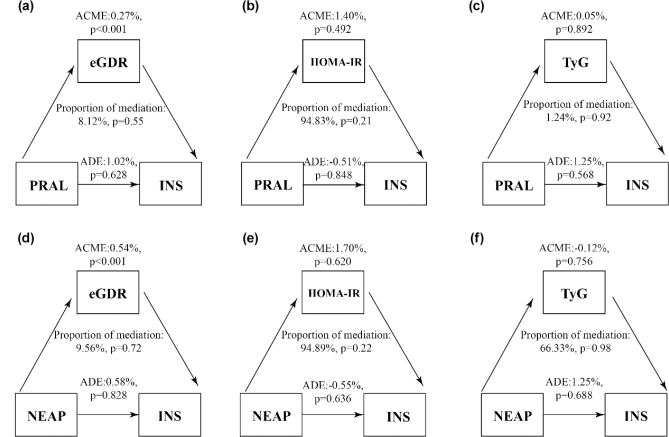
Analysis of mediating effects. (a) The mediating effect of eGDR between PRAL and fasting insulin was 8.12%, which was not significant. (b) The mediating effect of HOMA-IR in PRAL and fasting insulin was 94.83%, which was not significant. (c) The mediating effect of TyG in PRAL and fasting insulin was 1.24%, which was not significant. (d) The mediating effect of eGDR between NEAP and fasting insulin was 9.56%, which was not significant. (e) The mediating effect of HOMA-IR in NEAP and fasting insulin was 94.89%, which was not significant. (f) The mediating effect of TyG in NEAP and fasting insulin was 66.33%, which was not significant.

Furthermore, as illustrated in Fig. 3, neither eGDR, HOMA-IR, nor the TyG index demonstrated a significant mediating role in the relationship between DAL and fasting insulin (INS) levels.

### The RCS curve between DAL and the glucose metabolism indicators of the patient population

**Fig. 4:**
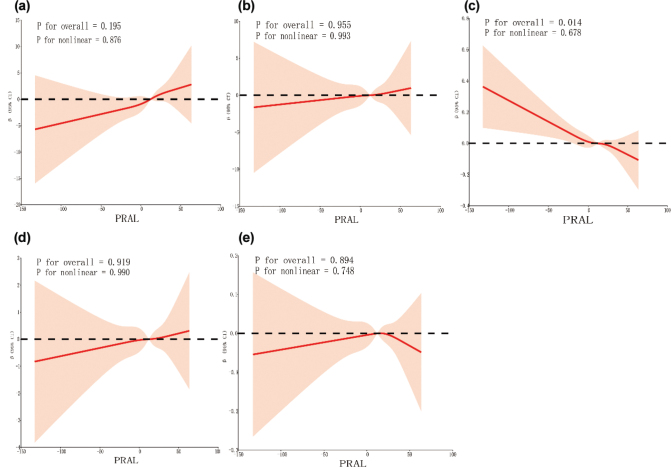
Restricted cubic spline regression analysis. (a) There is no nonlinear relationship between PRAL and fasting blood glucose. (b) There is no nonlinear relationship between PRAL and fasting insulin. (c) There is no nonlinear relationship between PRAL and eGDR. (d) There is no nonlinear relationship between PRAL and HOMA-IR. (e) There is no nonlinear relationship between PRAL and TyG.

**Fig. 5:**
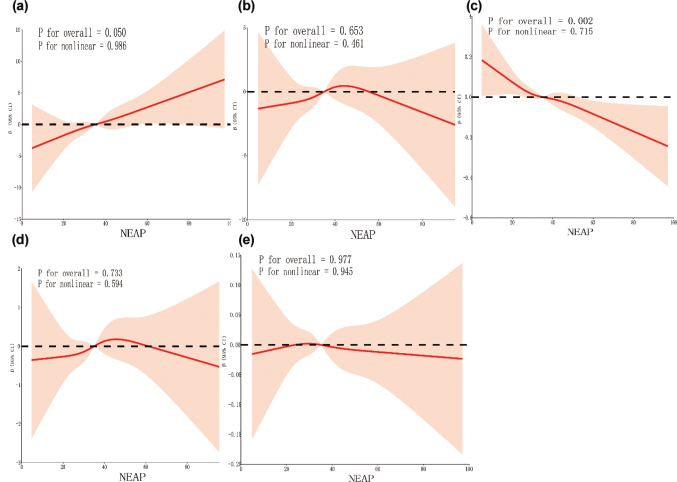
Restricted cubic spline regression analysis. (a) There is no nonlinear relationship between NEAP and fasting blood glucose. (b) There is no nonlinear relationship between NEAP and fasting insulin. (c) There is no nonlinear relationship between NEAP and eGDR. (d) There is no nonlinear relationship between NEAP and HOMA-IR. (e) There is no nonlinear relationship between NEAP and TyG.

Figures 4 and 5 present the RCS curves illustrating the relationships of PRAL and NEAP with fasting blood glucose, fasting insulin, eGDR, HOMA-IR, and the TyG index. No significant nonlinear associations were observed between these variables (*P* for nonlinearity > 0.05).

### Sensitivity analysis and NHANES comparison

In Supplementary File 1, we analyzed the association between DAL and glucose metabolism indicators in the overall population (Supplementary Tables 7–16). Continuous DAL measures remained significantly associated with fasting blood glucose and eGDR (*P* < 0.05), with effect directions consistent with those observed in the population with dysglycemia. In contrast, no significant associations were observed between DAL and fasting insulin, TyG index, or HOMA-IR. It is worth noting that the highest quartile of DAL (Q4) showed partial or full significant associations with most indicators, except for the TyG index. Additionally, significant nonlinear relationships were identified between PRAL and both fasting blood glucose and eGDR (Supplementary Fig. 1).

In Supplementary File 2, we present the results of the analysis examining the influence of DAL on diabetes, prediabetes, and glucose metabolism indicators in the NHANES population. As shown in Supplementary Tables 18 and 19, no significant association was observed between DAL and either diabetes or prediabetes, and the direction of the point estimates was opposite to that in the Chinese population (e.g. PRAL: OR 0.989, 95% CI: 0.983, 0.996). Furthermore, unlike in the Chinese cohort, DAL was not significantly associated with fasting blood glucose in the U.S. population (Supplementary Tables 22 and 23; *P* > 0.05), indicating differences between the two populations. Although logistic regression yielded opposite trends to those in the Chinese population and no significant mediation effects were detected (Supplementary Figs. 3 and 4), higher DAL levels were still associated with elevated fasting insulin, reduced eGDR, and higher HOMA-IR in some partially adjusted models analyzing glucose metabolism. In the analysis of the full NHANES population (Supplementary Tables 30–39), the results were consistent with those in the dysglycemia subgroup, showing no significant correlations between DAL and any of the glucose metabolism indicators.

## Discussion

This study represents the first large-scale cross-sectional investigation examining the association between DAL and diabetes and glucose metabolism indicators among Chinese adults. The analysis utilized comprehensive data from a geographically diverse population and adjusted for a wide range of potential confounding factors. Our findings demonstrate that both PRAL and NEAP are significantly associated with an increased risk of diabetes and prediabetes in the Chinese population. Higher DAL levels were correlated with elevated fasting blood glucose and reduced eGDR in individuals with dysglycemia. Moreover, eGDR was identified as a significant mediator in the relationship between DAL and fasting glucose levels.

The influence of diet on acid‑base balance is of vital importance. Fruits and vegetables are rich in potassium salts of metabolizable anions, which have an alkalizing effect. In contrast, sulfur amino acids contained in animal proteins and grains are nonmetabolizable anions and are decisive factors for daily acid load (30–32). People who mainly consume animal protein also have higher urine PH and excretion of uric acid, phosphate and other substances (32, 33). Meanwhile, other studies have also shown that a higher DAL may be associated with a higher risk of cancer (34). Moreover, a high DAL may be an unfavorable factor for cancer risk and prognosis (35). Meanwhile, high DAL is directly associated with an increased risk of CKD and decreased renal function (36), as well as independent risk factors for higher DAL blood pressure and elevated TG (37).

Dietary acid is a key factor influencing acid‑base balance in patients with chronic conditions. Currently, the most common methods for estimating acid‑base balance are PRAL and NEAP, which are calculated based on dietary components such as calcium, phosphorus, magnesium, potassium, and protein. This study is the first to identify a significant association between DAL and the prevalence of diabetes and prediabetes in the Chinese population. Specifically, higher DAL was correlated with elevated fasting blood glucose and reduced eGDR in individuals with dysglycemia, but no significant association was observed with fasting insulin, HOMA-IR, or the TyG. These findings align with and extend previous international research. For instance, a 7.4-year prospective Korean study linked higher DAL to an increased risk of future insulin resistance (38); an Australian cohort study suggested that insulin resistance related to long-term Western diet may be mediated by mild metabolic acidosis (39); an Iranian cross-sectional study reported an association between higher acid load and increased prevalence of prediabetes (40); a 16-week randomized controlled trial in overweight adults found positive correlations between changes in PRAL/NEAP and weight, fat mass, visceral fat, and insulin resistance (41). Our study not only fills a critical gap in evidence from China but also uniquely identifies eGDR as a significant mediator in the relationship between DAL and fasting glucose. This suggests that maintaining higher eGDR – a surrogate marker of insulin resistance derived from waist circumference, hypertension status, and HbA1c – may not only reduce the risk of stroke, cardiovascular events, and related mortality (27, 42–45) but also play a crucial role in stabilizing glucose fluctuations and improving long-term outcomes in individuals with diabetes or prediabetes.

Finally, sensitivity analysis in the overall Chinese population confirmed that the association between DAL and both fasting blood glucose and eGDR remained consistent and statistically significant. In contrast, cross-sectional analysis of the U.S. population using NHANES data showed no significant association between DAL and the prevalence of diabetes or prediabetes. Furthermore, the relationship between DAL and glucose metabolism indicators lacked consistent significance across statistical models, with associations observed only in a limited number of partially adjusted models for fasting glucose, fasting insulin, and eGDR. These findings are inconsistent with previous cohort studies (18). However, another NHANES-based study indicated that high dietary intake of carbohydrates, saturated fatty acids, monounsaturated fatty acids, polyunsaturated fatty acids, protein, total fat, and cholesterol was associated with impaired glucose tolerance (46). Thus, the relationship between DAL and diabetes or glucose metabolism in the U.S. population warrants further investigation through more detailed nutrient composition analyses and prospective cohort studies.

The pathophysiology of type 2 diabetes is initiated by the failure of β-cell function and insulin resistance in multiple tissues (47) and gradually spreads to the dysfunction of multiple organs such as the kidneys, brain, gastrointestinal tract, and adipose tissue, accompanied by chronic inflammation and immune dysregulation, resulting in a systemic, networked metabolic syndrome (48). The underlying mechanisms linking DAL to blood glucose dysregulation and insulin resistance have not been fully elucidated, but several plausible explanations have been proposed. Diets high in acid-forming animal-based foods such as meat, fish, and cheese can increase endogenous acid production (49). Such dietary patterns, which are rich in animal products, have frequently been associated with various metabolic disorders and inflammatory processes (50, 51), particularly among obese individuals (52). Inflammatory cytokines released during these processes may directly impair insulin sensitivity and contribute to abnormal glucose tolerance (53). Furthermore, chronic hyperglycemia and insulin resistance can promote the accumulation of visceral adipose tissue (54), creating a vicious cycle that exacerbates lipogenesis and ultimately leads to insulin resistance-related diabetes (55–57).

Our study has several limitations that should be acknowledged. First, as a cross-sectional investigation, it cannot establish a causal relationship between DAL and diabetes or glucose metabolism indicators. Second, the PRAL and NEAP scores were derived from mathematical model formulas rather than objective physiological measurements. Third, dietary assessment relied on self-reported questionnaires, which are inherently subject to recall bias. Finally, due to practical constraints, genetic factors were not considered in this analysis. Future research should seek to incorporate genetic polymorphisms to provide more individualized and precise strategies for diabetes prevention and treatment.

## Conclusion

In summary, our findings indicate that a higher DAL is associated with an increased prevalence of diabetes and prediabetes, as well as elevated fasting blood glucose and reduced eGDR among affected individuals. These results highlight the clinical importance of DAL management in diabetes prevention and glycemic control, offering valuable insights for improving long-term health outcomes in this population.

## Supplementary Material



## Data Availability

Online website (https://chns.cpc.unc.edu/data/) contains the datasets used in this investigation. When registration is reviewed and approved, the data set could be downloaded following the provided instructions. Data used in this study from 2011 to 2016 National Health and Nutrition Examination Survey (NHANES) data sets can be accessed through the NHANES website: https://www.cdc.gov/nchs/nhanes/index.htm.
