# Supplementary material for: Association of dietary acid load with diabetes and glucose metabolism index in Chinese adults: a cross-sectional study
Source: Food Nutr Res. 2026 Mar 20;70:13470. doi: 10.29219/fnr.v70.13470 (PMC13054938; doi:10.29219/fnr.v70.13470)
Supplement: Supplementary file 1 [file FNR-70-13470-s1.docx]

**1.** **The relationship between DAL and other glucose metabolism indicators in the patient population**

**TableS1**. The association between PRAL and fasting insulin

|  | **Model1** | **Model2** | **Model3** | **Model4** |
| --- | --- | --- | --- | --- |
| **Continuous** | 0.012(-0.029,0.053)  0.568 | 0.013(-0.029,0.055)  0.540 | 0.008(-0.033,0.050)  0.686 | 0.012(-0.030,0.054)  0.577 |
| **Q1** | **Ref** | **Ref** | **Ref** | **Ref** |
| **Q2** | 1.245(-1.279,3.769)  0.334 | 1.222(-1.305,3.749)  0.343 | 1.037(-1.468,3.542)  0.417 | 0.974(-1.514,3.488)  0.448 |
| **Q3** | 0.345(-2.179,2.870)  0.789 | 0.375(-2.158,2.909)  0.772 | 0.190(-2.320,2.699)  0.882 | 0.199(-2.314,2.711)  0.877 |
| **Q4** | 0.453(-2.070,2.976)  0.725 | 0.500(-2.068,3.067)  0.703 | 0.108(-2.437,2.654)  0.934 | 0.333(-2.311,2.976)  0.805 |

Model1: No covariates were adjusted.

Model2: Adjusted for age, sex, education and place.

Model3: Adjusted for age, sex, education, place, BMI, hypertension, myocardial infarct, stroke, chronic kidney disease, hyperuricemia and smoke status.

Model4: Adjusted for age, sex, education, place, BMI, hypertension, myocardial infarct, stroke, chronic kidney disease, hyperuricemia, smoke status, calorie, carbohydrate and fat.

**TableS2**. The association between NEAP and fasting insulin

|  | **Model1** | **Model2** | **Model3** | **Model4** |
| --- | --- | --- | --- | --- |
| **Continuous** | 0.026(-0.041,0.093)  0.445 | 0.027(-0.040,0.094)  0.432 | 0.005(-0.062,0.072)  0.876 | 0.010(-0.058,0.077)  0.781 |
| **Q1** | **Ref** | **Ref** | **Ref** | **Ref** |
| **Q2** | 1.079(-1.446,3.603)  0.402 | 1.020(-1.511,3.550)  0.430 | 0.523(-1.987,3.032)  0.683 | 0.565(-1.946,3.077)  0.659 |
| **Q3** | 1.079(-0.775,4.274)  0.174 | 1.787(-0.752,4.327)  0.168 | 1.142(-1.037,3.999)  0.249 | 1.549(-0.977,4.075)  0.229 |
| **Q4** | 1.761(-0.763,4.286)  0.172 | 1.814(-0.722,4.349)  0.161 | 1.179(-1.138,3.695)  0.359 | 1.360(-1.173,3.893)  0.293 |

Model1: No covariates were adjusted.

Model2: Adjusted for age, sex, education and place.

Model3: Adjusted for age, sex, education, place, BMI, hypertension, myocardial infarct, stroke, chronic kidney disease, hyperuricemia and smoke status.

Model4: Adjusted for age, sex, education, place, BMI, hypertension, myocardial infarct, stroke, chronic kidney disease, hyperuricemia, smoke status, calorie, carbohydrate and fat.

**TableS3**. The association between PRAL and TyG

|  | **Model1** | **Model2** | **Model3** | **Model4** |
| --- | --- | --- | --- | --- |
| **Continuous** | 0.000(-0.001,0.001)  0.752 | 0.000(-0.001,0.001)  0.928 | -0.000(-0.001,0.001)  0.887 | -0.000(-0.001,0.001)  0.865 |
| **Q1** | **Ref** | **Ref** | **Ref** | **Ref** |
| **Q2** | -0.021(-0.089,0.046)  0.534 | -0.026(-0.093,0.042)  0.457 | -0.008(-0.068,-0.052)  0.799 | -0.013(-0.073,0.047)  0.660 |
| **Q3** | 0.014(-0.054,0.081)  0.692 | 0.010(-0.058,0.077)  0.778 | 0.015(-0.045,0.075)  0.623 | 0.017(-0.043,0.077)  0.582 |
| **Q4** | 0.004(-0.063,0.072)  0.900 | -0.004(-0.072,0.064)  0.905 | -0.017(-0.078,0.044)  0.586 | 0.001(-0.062,0.064)  0.970 |

Model1: No covariates were adjusted.

Model2: Adjusted for age, sex, education and place.

Model3: Adjusted for age, sex, education, place, BMI, hypertension, myocardial infarct, stroke, chronic kidney disease, hyperuricemia and smoke status.

Model4: Adjusted for age, sex, education, place, BMI, hypertension, myocardial infarct, stroke, chronic kidney disease, hyperuricemia, smoke status, calorie, carbohydrate and fat.

**TableS4**. The association between NEAP and TyG

|  | **Model1** | **Model2** | **Model3** | **Model4** |
| --- | --- | --- | --- | --- |
| **Continuous** | 0.001(-0.001,0.003)  0.209 | 0.001(-0.001,0.003)  0.280 | -0.000(-0.002,0.001)  0.885 | -0.000(-0.002,0.001)  0.761 |
| **Q1** | **Ref** | **Ref** | **Ref** | **Ref** |
| **Q2** | 0.031(-0.037,0.098)  0.370 | 0.024(-0.044,0.091)  0.491 | 0.013(-0.047,0.073)  0.678 | 0.013(-0.046,0.073)  0.660 |
| **Q3** | 0.019(-0.048,0.087)  0.572 | 0.012(-0.055,0.080)  0.725 | 0.016(-0.044,0.076)  0.598 | 0.018(-0.042,0.078)  0.555 |
| **Q4** | -0.003(-0.070,0.065)  0.933 | -0.007(-0.075,0.060)  0.830 | -0.020(-0.080,0.040)  0.518 | -0.023(-0.084,0.037)  0.451 |

Model1: No covariates were adjusted.

Model2: Adjusted for age, sex, education and place.

Model3: Adjusted for age, sex, education, place, BMI, hypertension, myocardial infarct, stroke, chronic kidney disease, hyperuricemia and smoke status.

Model4: Adjusted for age, sex, education, place, BMI, hypertension, myocardial infarct, stroke, chronic kidney disease, hyperuricemia, smoke status, calorie, carbohydrate and fat.

**TableS5**. The association between PRAL and HOMA-IR

|  | **Model1** | **Model2** | **Model3** | **Model4** |
| --- | --- | --- | --- | --- |
| **Continuous** | -0.001(-0.004,0.001)  0.297 | 0.005(-0.009,0.019)  0.471 | 0.003(-0.011,0.017)  0.630 | 0.005(-0.009,0.019)  0.489 |
| **Q1** | **Ref** | **Ref** | **Ref** | **Ref** |
| **Q2** | 0.560(-0.297,1.416)  0.200 | 0.547(-0.310,1.405)  0.211 | 0.469(-0.379,1.318)  0.278 | 0.438(-0.414,1.289)  0.314 |
| **Q3** | 0.277(-0.580,1.134)  0.526 | 0.290(-0.569,1.150)  0.508 | 0.214(-0.636,1.064)  0.621 | 0.223(-0.628,1.074)  0.607 |
| **Q4** | 0.130(-0.726,0.986)  0.766 | 0.147(-0.724,1.018)  0.740 | -0.003(-0.866,0.859)  0.994 | 0.107(-0.788,1.003)  0.815 |

Model1: No covariates were adjusted.

Model2: Adjusted for age, sex, education and place.

Model3: Adjusted for age, sex, education, place, BMI, hypertension, myocardial infarct, stroke, chronic kidney disease, hyperuricemia and smoke status.

Model4: Adjusted for age, sex, education, place, BMI, hypertension, myocardial infarct, stroke, chronic kidney disease, hyperuricemia, smoke status, calorie, carbohydrate and fat.

**TableS6**. The association between NEAP and HOMA-IR

|  | **Model1** | **Model2** | **Model3** | **Model4** |
| --- | --- | --- | --- | --- |
| **Continuous** | 0.013(-0.010,0.035)  0.272 | 0.013(-0.010,0.035)  0.280 | 0.005(-0.018,0.027)  0.681 | 0.006(-0.017,0.029)  0.623 |
| **Q1** | **Ref** | **Ref** | **Ref** | **Ref** |
| **Q2** | 0.293(-0.563,1.150)  0.502 | 0.279(-0.580,1.138)  0.524 | 0.089(-0.762,0.939)  0.838 | 0.105(-0.746,0.956)  0.809 |
| **Q3** | 0.417(-0.439,1.274)  0.340 | 0.419(-0.442,1.281)  0.340 | 0.296(-0.557,1.149)  0.497 | 0.326(-0.530,1.182)  0.455 |
| **Q4** | 0.710(-0.147,1.567)  0.104 | 0.724(-0.136,1.584)  0.099 | 0.484(-0.368,1.337)  0.266 | 0.532(-0.326,1.391)  0.224 |

Model1: No covariates were adjusted.

Model2: Adjusted for age, sex, education and place.

Model3: Adjusted for age, sex, education, place, BMI, hypertension, myocardial infarct, stroke, chronic kidney disease, hyperuricemia and smoke status.

Model4: Adjusted for age, sex, education, place, BMI, hypertension, myocardial infarct, stroke, chronic kidney disease, hyperuricemia, smoke status, calorie, carbohydrate and fat.

**2.The association between DAL and glucose metabolism indicators in the overall population**

**TableS7**. The association between PRAL and fasting insulin

|  | **Model1** | **Model2** | **Model3** | **Model4** |
| --- | --- | --- | --- | --- |
| **Continuous** | 0.020(-0.002,0.042)  0.068 | 0.022(-0.000,0.044)  0.052 | 0.017(-0.005,0.039)  0.122 | 0.019(-0.003,0.041)  0.083 |
| **Q1** | **Ref** | **Ref** | **Ref** | **Ref** |
| **Q2** | 0.418(-0.961,1.797)  0.553 | 0.393(-0.987,1.772)  0.577 | 0.417(-0.946,1.780)  0.549 | 0.337(-1.029,1.704)  0.628 |
| **Q3** | 0.078(-1.301,1.457)  0.912 | 0.128(-1.254,1.509)  0.856 | 0.073(-1.291,1.438)  0.916 | 0.073(-1.293,1.438)  0.917 |
| **Q4** | **1.445(0.066,2.842)**  **0.040** | **1.580(0.179,2.981)**  **0.027** | 1.234(-0.150,2.691)  0.081 | **1.549(0.119,2.980)**  **0.034** |

Model1: No covariates were adjusted.

Model2: Adjusted for age, sex, education and place.

Model3: Adjusted for age, sex, education, place, BMI, hypertension, myocardial infarct, stroke, chronic kidney disease, hyperuricemia and smoke status.

Model4: Adjusted for age, sex, education, place, BMI, hypertension, myocardial infarct, stroke, chronic kidney disease, hyperuricemia, smoke status, calorie, carbohydrate and fat.

**TableS8**. The association between NEAP and fasting insulin

|  | **Model1** | **Model2** | **Model3** | **Model4** |
| --- | --- | --- | --- | --- |
| **Continuous** | **0.046(0.009,0.084)**  **0.014** | **0.046(0.009,0.083)**  **0.016** | 0.030(-0.006,0.067)  0.105 | 0.032(-0.005,0.069)  0.091 |
| **Q1** | **Ref** | **Ref** | **Ref** | **Ref** |
| **Q2** | 0.443(-0.937,1.822)  0.529 | 0.420(-0.962,1.803)  0.551 | 0.362(-1.003,1.782)  0.603 | 0.391(-0.976,1.758)  0.575 |
| **Q3** | 1.054(-0.324,2.432)  0.134 | 1.068(-0.314,2.451)  0.130 | 1.001(-0.365,2.368)  0.151 | 1.051(-0.319,2.422)  0.133 |
| **Q4** | **2.053(0.656,3.413)**  **0.004** | **2.061(0.675,3.446)**  **0.004** | **1.673(0.303,3.044)**  **0.017** | **1.766(0.386,3.146)**  **0.012** |

Model1: No covariates were adjusted.

Model2: Adjusted for age, sex, education and place.

Model3: Adjusted for age, sex, education, place, BMI, hypertension, myocardial infarct, stroke, chronic kidney disease, hyperuricemia and smoke status.

Model4: Adjusted for age, sex, education, place, BMI, hypertension, myocardial infarct, stroke, chronic kidney disease, hyperuricemia, smoke status, calorie, carbohydrate and fat.

**TableS9**. The association between PRAL and fasting blood glucose

|  | **Model1** | **Model2** | **Model3** | **Model4** |
| --- | --- | --- | --- | --- |
| **Continuous** | **0.042(0.017,0.067)**  **0.001** | **0.051(0.026,0.076)**  **<0.001** | **0.044(0.020,0.069)**  **<0.001** | **0.042(0.018,0.067)**  **<0.001** |
| **Q1** | **Ref** | **Ref** | **Ref** | **Ref** |
| **Q2** | **1.580(-0.030,3.190)**  **0.055** | 1.500(-0.082,3.082)  0.063 | **1.676(0.126,3.266)**  **0.034** | 1.466(-0.083,3.016)  0.064 |
| **Q3** | **1.841(0.231,3.451)**  **0.025** | **2.154(0.570,3.739)**  **0.008** | **2.122(0.571,3.674)**  **0.007** | **2.057(0.509,3.605)**  **0.009** |
| **Q4** | **2.723(1.113,4.333)**  **<0.001** | **3.307(1.700,4.913)**  **<0.001** | **2.853(1.278,4.428)**  **<0.001** | **3.016(1.395,4.638)**  **<0.001** |

Model1: No covariates were adjusted.

Model2: Adjusted for age, sex, education and place.

Model3: Adjusted for age, sex, education, place, BMI, hypertension, myocardial infarct, stroke, chronic kidney disease, hyperuricemia and smoke status.

Model4: Adjusted for age, sex, education, place, BMI, hypertension, myocardial infarct, stroke, chronic kidney disease, hyperuricemia, smoke status, calorie, carbohydrate and fat.

**TableS10**. The association between NEAP and fasting blood glucose

|  | **Model1** | **Model2** | **Model3** | **Model4** |
| --- | --- | --- | --- | --- |
| **Continuous** | **0.124(0.081,0.168)**  **<0.001** | **0.126(0.083,0.168)**  **<0.001** | **0.109(0.067,0.151)**  **<0.001** | **0.099(0.057,0.142)**  **<0.001** |
| **Q1** | **Ref** | **Ref** | **Ref** | **Ref** |
| **Q2** | 1.462(-0.149,3.072)  0.075 | **1.687(0.102,3.272)**  **0.037** | **1.729(0.176,3.282)**  **0.029** | **1.589(0.039,3.138)**  **0.044** |
| **Q3** | **2.021(0.412,3.630)**  **0.014** | **2.172(0.587,3.758)**  **0.007** | **2.193(0.639,3.746)**  **0.006** | **2.099(0.545,3.653)**  **0.008** |
| **Q4** | **3.492(1.883,5.102)**  **<0.001** | **3.759(2.171,5.348)**  **<0.001** | **3.409(1.851,4.967)**  **<0.001** | **3.058(1.494,4.622)**  **<0.001** |

Model1: No covariates were adjusted.

Model2: Adjusted for age, sex, education and place.

Model3: Adjusted for age, sex, education, place, BMI, hypertension, myocardial infarct, stroke, chronic kidney disease, hyperuricemia and smoke status.

Model4: Adjusted for age, sex, education, place, BMI, hypertension, myocardial infarct, stroke, chronic kidney disease, hyperuricemia, smoke status, calorie, carbohydrate and fat.

**TableS11**. The association between PRAL and eGDR

|  | **Model1** | **Model2** | **Model3** | **Model4** |
| --- | --- | --- | --- | --- |
| **Continuous** | -0.001(-0.003,0.000)  0.160 | **-0.003(-0.99, -0.001)**  **<0.001** | **-0.002(-0.99, -0.001)**  **<0.001** | **-0.002(-0.99, -0.001)**  **0.002** |
| **Q1** | **Ref** | **Ref** | **Ref** | **Ref** |
| **Q2** | 0.025(-0.086,0.136)  0.660 | 0.035(-0.069,0.139)  0.510 | **-0.064(-0.111, -0.018)**  **0.007** | **-0.065(-0.122, -0.018)**  **0.007** |
| **Q3** | 0.040(-0.071,0.152)  0.479 | -0.011(-0.115,0.093)  0.837 | -0.024(-0.071,0.023)  0.311 | -0.022(-0.069, 0.025)  0.352 |
| **Q4** | 0.004(-0.108,0.115)  0.945 | -0.105(-0.211,0.000)  0.051 | **-0.070(-0.118, -0.022)**  **0.004** | **-0.060(-0.109, -0.011)**  **0.016** |

Model1: No covariates were adjusted.

Model2: Adjusted for age, sex, education and place.

Model3: Adjusted for age, sex, education, place, BMI, hypertension, myocardial infarct, stroke, chronic kidney disease, hyperuricemia and smoke status.

Model4: Adjusted for age, sex, education, place, BMI, hypertension, myocardial infarct, stroke, chronic kidney disease, hyperuricemia, smoke status, calorie, carbohydrate and fat.

**TableS12**. The association between NEAP and eGDR

|  | **Model1** | **Model2** | **Model3** | **Model4** |
| --- | --- | --- | --- | --- |
| **Continuous** | **-0.005(-0.99, -0.002)**  **0.001** | **-0.005(-0.99, -0.003)**  **<0.001** | **-0.003(-0.99, -0.001)**  **<0.001** | **-0.002(-0.99, -0.001)**  **<0.001** |
| **Q1** | **Ref** | **Ref** | **Ref** | **Ref** |
| **Q2** | 0.041(-0.071,0.152)  0.473 | 0.002(-0.102,0.106)  0.971 | -0.026(-0.073,0.021)  0.272 | -0.024(-0.071,0.023)  0.326 |
| **Q3** | 0.038(-0.073,0.150)  0.499 | 0.000(-0.104,0.005)  0.993 | -0.045(-0.092,0.002)  0.059 | -0.041(-0.088, 0.007)  0.091 |
| **Q4** | -0.075(-0.186,0.037)  0.188 | **-0.129(-0.233,-0.025)**  **0.015** | **-0.061(-0.109, -0.014)**  **0.011** | **-0.054(-0.101, -0.006)**  **0.026** |

Model1: No covariates were adjusted.

Model2: Adjusted for age, sex, education and place.

Model3: Adjusted for age, sex, education, place, BMI, hypertension, myocardial infarct, stroke, chronic kidney disease, hyperuricemia and smoke status.

Model4: Adjusted for age, sex, education, place, BMI, hypertension, myocardial infarct, stroke, chronic kidney disease, hyperuricemia, smoke status, calorie, carbohydrate and fat.

**TableS13**. The association between PRAL and TyG

|  | **Model1** | **Model2** | **Model3** | **Model4** |
| --- | --- | --- | --- | --- |
| **Continuous** | 0.000(-0.000,0.001)  0.234 | 0.001(-0.000,0.001)  0.146 | 0.000(-0.000,0.001)  0.563 | 0.000(-0.000,0.001)  0.439 |
| **Q1** | **Ref** | **Ref** | **Ref** | **Ref** |
| **Q2** | -0.017(-0.061,0.027)  0.452 | -0.019(-0.063,0.025)  0.398 | -0.003(-0.042,0.036)  0.876 | - 0.009(-0.048,0.029)  0.634 |
| **Q3** | 0.013(-0.031,0.058)  0.551 | 0.018(-0.026,0.062)  0.420 | 0.022(-0.017,0.061)  0.271 | 0.020(-0.018,0.059)  0.304 |
| **Q4** | 0.012(-0.033,0.056)  0.606 | 0.018(-0.027,0.062)  0.438 | -0.006(-0.045,0.033)  0.770 | 0.006(-0.034,0.047)  0.757 |

Model1: No covariates were adjusted.

Model2: Adjusted for age, sex, education and place.

Model3: Adjusted for age, sex, education, place, BMI, hypertension, myocardial infarct, stroke, chronic kidney disease, hyperuricemia and smoke status.

Model4: Adjusted for age, sex, education, place, BMI, hypertension, myocardial infarct, stroke, chronic kidney disease, hyperuricemia, smoke status, calorie, carbohydrate and fat.

**TableS14**. The association between NEAP and TyG

|  | **Model1** | **Model2** | **Model3** | **Model4** |
| --- | --- | --- | --- | --- |
| **Continuous** | 0.001(-0.001,0.003)  0.209 | 0.001(-0.000,0.002)  0.137 | 0.000(-0.001,0.001)  0.953 | -0.000(-0.001,0.001)  0.859 |
| **Q1** | **Ref** | **Ref** | **Ref** | **Ref** |
| **Q2** | 0.000(-0.044,0.044)  0.996 | 0.001(-0.043,0.045)  0.958 | 0.006(-0.033,0.045)  0.755 | 0.004(-0.034,0.043)  0.824 |
| **Q3** | 0.015(-0.029,0.059)  0.502 | 0.015(-0.029,0.058)  0.511 | 0.022(-0.017,0.061)  0.261 | 0.022(-0.017,0.061)  0.262 |
| **Q4** | 0.008(-0.036,0.052)  0.727 | 0.008(-0.035,0.052)  0.705 | -0.004(-0.043,0.034)  0.822 | -0.008(-0.047,0.031)  0.691 |

Model1: No covariates were adjusted.

Model2: Adjusted for age, sex, education and place.

Model3: Adjusted for age, sex, education, place, BMI, hypertension, myocardial infarct, stroke, chronic kidney disease, hyperuricemia and smoke status.

Model4: Adjusted for age, sex, education, place, BMI, hypertension, myocardial infarct, stroke, chronic kidney disease, hyperuricemia, smoke status, calorie, carbohydrate and fat.

**TableS15**. The association between PRAL and HOMA-IR

|  | **Model1** | **Model2** | **Model3** | **Model4** |
| --- | --- | --- | --- | --- |
| **Continuous** | 0.006(-0.001,0.013)  0.075 | **0.007(0.001,0.014)**  **0.044** | 0.006(-0.001,0.012)  0.113 | 0.006(-0.001,0.013)  0.076 |
| **Q1** | **Ref** | **Ref** | **Ref** | **Ref** |
| **Q2** | 0.218(-0.222,0.658)  0.331 | 0.208(-0.231,0.647)  0.353 | 0.213(-0.221,0.646)  0.336 | 0.182(-0.253,0.616)  0.412 |
| **Q3** | 0.086(-0.353,0.526)  0.700 | 0.115(-0.325,0.555)  0.609 | 0.090(-0.344,0.524)  0.684 | 0.088(-0.345,0.522)  0.689 |
| **Q4** | 0.420(-0.020,0.859)  0.061 | **0.480(0.034,0.926)**  **0.035** | 0.357(-0.083,0.798)  0.111 | **0.458(0.004,0.913)**  **0.048** |

Model1: No covariates were adjusted.

Model2: Adjusted for age, sex, education and place.

Model3: Adjusted for age, sex, education, place, BMI, hypertension, myocardial infarct, stroke, chronic kidney disease, hyperuricemia and smoke status.

Model4: Adjusted for age, sex, education, place, BMI, hypertension, myocardial infarct, stroke, chronic kidney disease, hyperuricemia, smoke status, calorie, carbohydrate and fat.

**TableS16**. The association between NEAP and HOMA-IR

|  | **Model1** | **Model2** | **Model3** | **Model4** |
| --- | --- | --- | --- | --- |
| **Continuous** | **0.016(0.005,0.028)**  **0.006** | **0.016(0.005,0.028)**  **0.007** | 0.011(-0.001,0.023)  0.061 | 0.011(-0.000,0.023)  0.057 |
| **Q1** | **Ref** | **Ref** | **Ref** | **Ref** |
| **Q2** | 0.099(-0.341,0.538)  0.660 | 0.279(-0.580,1.138)  0.633 | 0.084(-0.350,0.518)  0.706 | 0.089(-0.346,0.523)  0.690 |
| **Q3** | 0.257(-0.182,0.696)  0.252 | 0.419(-0.442,1.281)  0.231 | 0.240(-0.194,0.674)  0.278 | 0.255(-0.180,0.691)  0.251 |
| **Q4** | **0.651(0.211,1.090)**  **0.004** | **0.724(-0.136,1.584)**  **0.003** | **0.537(0.102,0.973)**  **0.016** | **0.556(0.118,0.995)**  **0.013** |

Model1: No covariates were adjusted.

Model2: Adjusted for age, sex, education and place.

Model3: Adjusted for age, sex, education, place, BMI, hypertension, myocardial infarct, stroke, chronic kidney disease, hyperuricemia and smoke status.

Model4: Adjusted for age, sex, education, place, BMI, hypertension, myocardial infarct, stroke, chronic kidney disease, hyperuricemia, smoke status, calorie, carbohydrate and fat.

**3.** **The RCS curve between DAL and the glucose metabolism indicators of the overall population**

**FigS1.**


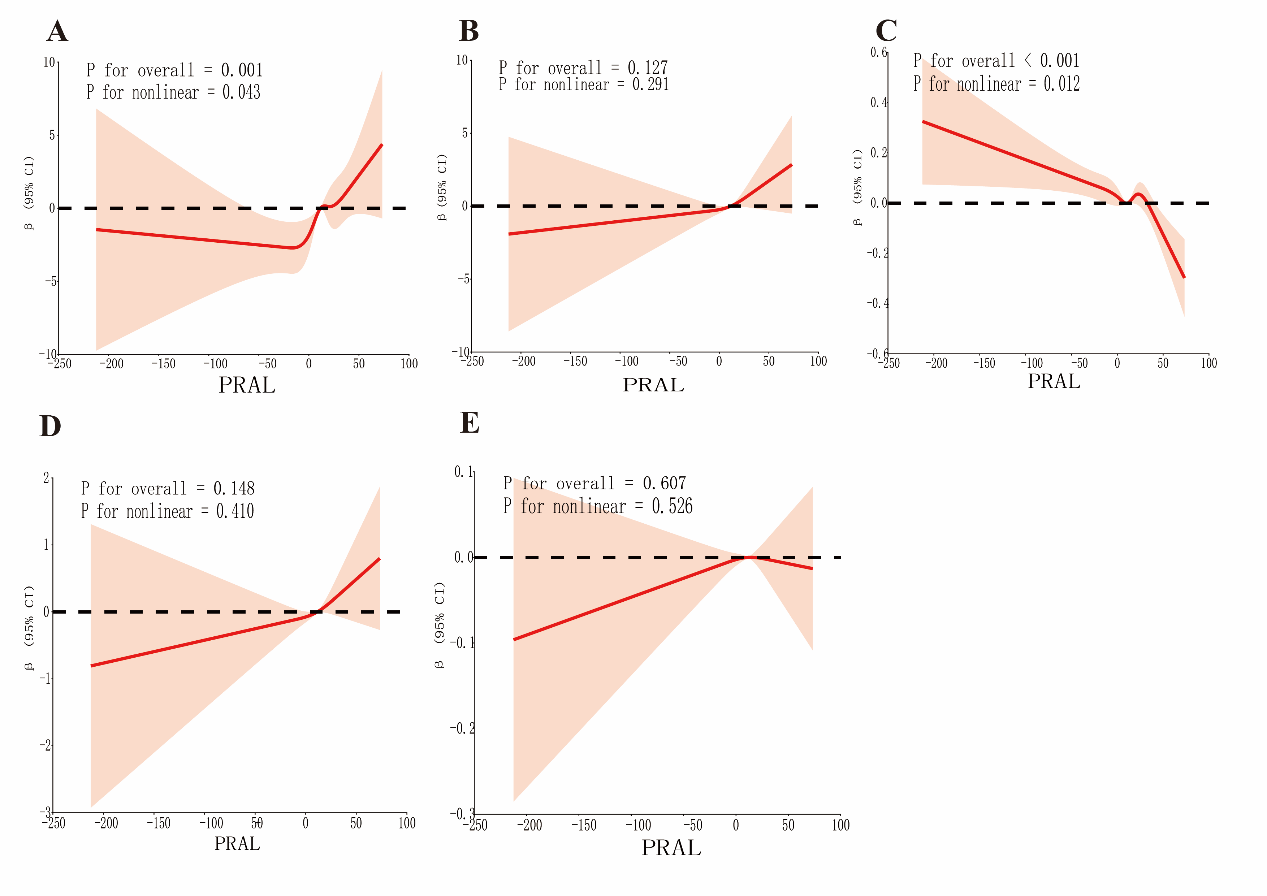


Fig.S1 Restricted cubic spline regression analysis. (A) There is no nonlinear relationship between PRAL and fasting blood glucose; (B) There is no nonlinear relationship between PRAL and fasting insulin. (C) There is a significant nonlinear relationship between PRAL and eGDR. (D) There is no nonlinear relationship between PRAL and HOMA-IR. (E) There is no nonlinear relationship between PRAL and TyG.

**FigS2.
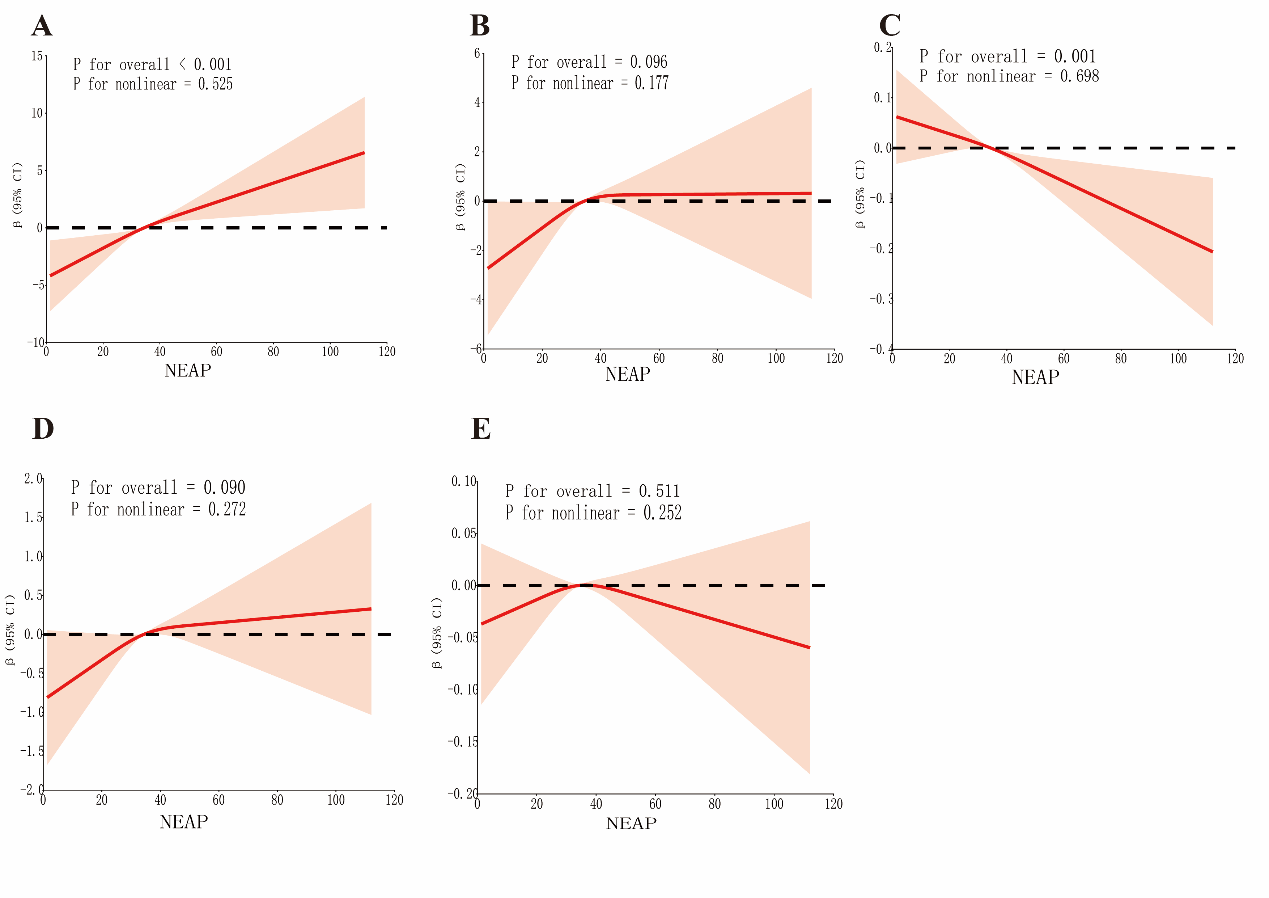
**

Fig.S2 Restricted cubic spline regression analysis. (A) There is no nonlinear relationship between PRAL and fasting blood glucose; (B) There is no nonlinear relationship between PRAL and fasting insulin. (C) There is no nonlinear relationship between PRAL and eGDR. (D) There is no nonlinear relationship between PRAL and HOMA-IR. (E) There is no nonlinear relationship between PRAL and TyG.
